# NECKCHECK PROJECT: enhancing diagnostic accuracy in oropharyngeal squamous cell carcinoma through computer-based radiological tools

**DOI:** 10.1038/s41598-025-03895-8

**Published:** 2025-06-04

**Authors:** Sara María Ferrero-Coloma, Julián Izquierdo-Luzón, Avelino Pereira-Expósito, Jose Antonio Quesada, Carlos Ferrero-Coloma, Manuela Sancho-Mestre, Elena Garcia-Garrigós, Vicente Gil-Guillen

**Affiliations:** 1 Otolaryngology Department, Dr Balmis General University Hospital of Alicante, Alicante, Alicante Spain; 2 Clinical Medicine Department, Miguel Hernández University, San Juan de Alicante, Alicante Spain; 3 Medical Research Department, General University Hospital of Elda, Elda, Spain; 4 Anesthesiology Department, Dr. Balmis General University Hospital of Alicante, Alicante, Spain; 5 Radiology Department, Dr. Balmis General University Hospital of Alicante, Alicante, Spain; 6 Otolaryngology Department, General Hospital of Elda, Elda, Alicante Spain

**Keywords:** Head and neck neoplasm, Clinical protocols, Radiological image interpretation, Computer assisted, Medical informatics, Mobile app, Checklist, Oropharynx, Head and neck cancer, Oral cancer, Oncology, Surgical oncology

## Abstract

The interpretation of radiological images for head and neck tumors often lacks standardized protocols, increasing the risk of diagnostic inconsistencies. This study introduces a computerized radiological checklist designed to enhance diagnostic accuracy and standardize the evaluation of oropharyngeal squamous cell carcinoma (OPSCC) imaging among otolaryngologists (ENTs). A radiological checklist was developed based on a comprehensive literature review and digitized into an intuitive interface. A concordance study involving 15 ENTs was conducted, assessing 90 OPSCC cases in two phases: before and after using the checklist. Diagnostic agreement with radiologists was measured using Cohen’s kappa coefficient, and a mixed-effects linear model evaluated accuracy improvements, accounting for patient sex, age, stage, and HPV status. The checklist significantly improved diagnostic concordance, increasing Cohen’s kappa from 0.28 (95% CI: 0.09–0.46) without it to 0.66 (95% CI: 0.55–0.77) with it (*p* < 0.01). The mixed-effects model showed a mean improvement of 2.66 correct responses in the checklist group (SE 0.31, *p* < 0.001). This study supports the effectiveness of this checklist in improving the diagnostic consistency and accuracy of OPSCC imaging. This method shows promise as a practical tool to reduce errors and enhance clinical practice among ENT specialists.

**Supplementary Information:**

The online version contains supplementary material available at 10.1038/s41598-025-03895-8.

## Introduction

 Cancer incidence and mortality have been increasing rapidly in recent years, with the number of cases expected to reach 28.4 million worldwide in 2040, an increase of 47% compared with that in 2020^1^ The incidence of head and neck cancer (HNC) has also increased, driven mainly by new cases of oropharyngeal cancer linked to human papillomavirus (HPV) infections^2^.

The oropharynx has emerged as an area of special relevance in recent years, particularly because of its association with HPV, which accounts for more than 70% of oropharyngeal cancer cases in Europe and North America^3^A wide variety of tumors can appear in this region; however, the vast majority are squamous cell carcinomas (SCCs)^4^, the subject of this review. The oropharynx is anatomically defined by its anterior border with the oral cavity, its superior limit marked by the soft palate contiguous with the nasopharynx, and its posterior boundary extending to the vertebral column at the junction of the constrictors and caudal, delineated by an imaginary line passing through the hyoid^5^ Despite these delineations, the challenge lies in the intricate nature of tumor involvement, underscoring the pivotal role of radiology in diagnosis and management^6^.

This area is anatomically divided into distinct sublocations to facilitate systematic identification; however, the close proximity of these regions presents challenges in accurately localizing lesions on the basis solely of clinical symptoms and physical examination. Radiological assessments are essential for achieving precise localization. The four primary sublocations include the pharyngeal tonsils, soft palate, posterior wall, and base of the tongue^4^ Typically, magnetic resonance imaging (MRI) and computed tomography (CT) are preferred imaging modalities^6–9^ SCCs most commonly affect the pharyngeal tonsils, accounting for approximately 70–80% of cases, with a notable propensity for lymph node metastasis^10^ Although the soft palate is a small muscular bridge covered by mucosa, it lacks a true lateral barrier against tumor spread, resulting in a high likelihood of tumor dissemination. Involvement of the posterior pharyngeal wall, which exhibits biological behaviour similar to that of hypopharyngeal tumors, is rare^11^ Conversely, SCCs arising at the base of the tongue typically manifest in advanced stages because of their asymptomatic nature. Moreover, extensive crossed lymphatic drainage contributes to approximately 40% of patients presenting with contralateral cervical lymph node metastases^12^.

The proper selection and use of imaging studies is essential for the diagnosis and prognosis of patients with HNC. Specialist clinicians typically interpret radiological tests in their routine practice to consider specific data that may condition the patient’s treatment^13^ However, radiology training among physicians in other specialties, such as otolaryngologists, may be inadequate for accurate radiological diagnosis because of the lack of standardized protocols in residency programs^14^.

This project was designed to create a useful cognitive aid to systematize and protocolize the visualization of HNC in radiological images, helping to minimize avoidable errors among head and neck specialists such as otolaryngologists. Accordingly, the review and development of a system that encompasses the most critical concepts in oropharyngeal SCC (OPSCC) radiology may prove beneficial and potentially enhance diagnostic accuracy by refining the precision of image interpretation in patients with OPSCC. The primary objective of this study was to ascertain whether a radiological checklist, grounded in a comprehensive review of scientific evidence and integrated into a newly developed digital interface, enhances the interpretation of radiological images of patients with OPSCC by otolaryngology specialists (ENTs).

## Materials and methods

This study adhered to the ethical principles of the Helsinki Declaration and was approved by the Ethics Committee of the General University Hospital of Elda (protocol number 2022/55PI). It is also situated within a doctoral thesis program.

The project was structured into three separate phases. The first phase involved a comprehensive literature review focusing on the most significant concepts in image interpretation and their implications for both treatment planning and staging. In the second phase, these findings were standardized into a protocol, formalized as a checklist, and subsequently integrated into a digital interface designed as a freely accessible web application. The final phase consisted of the development of a study aimed at evaluating the concordance in image interpretation between radiologists and ENTs.

### Literature review

**Fig. 1:**
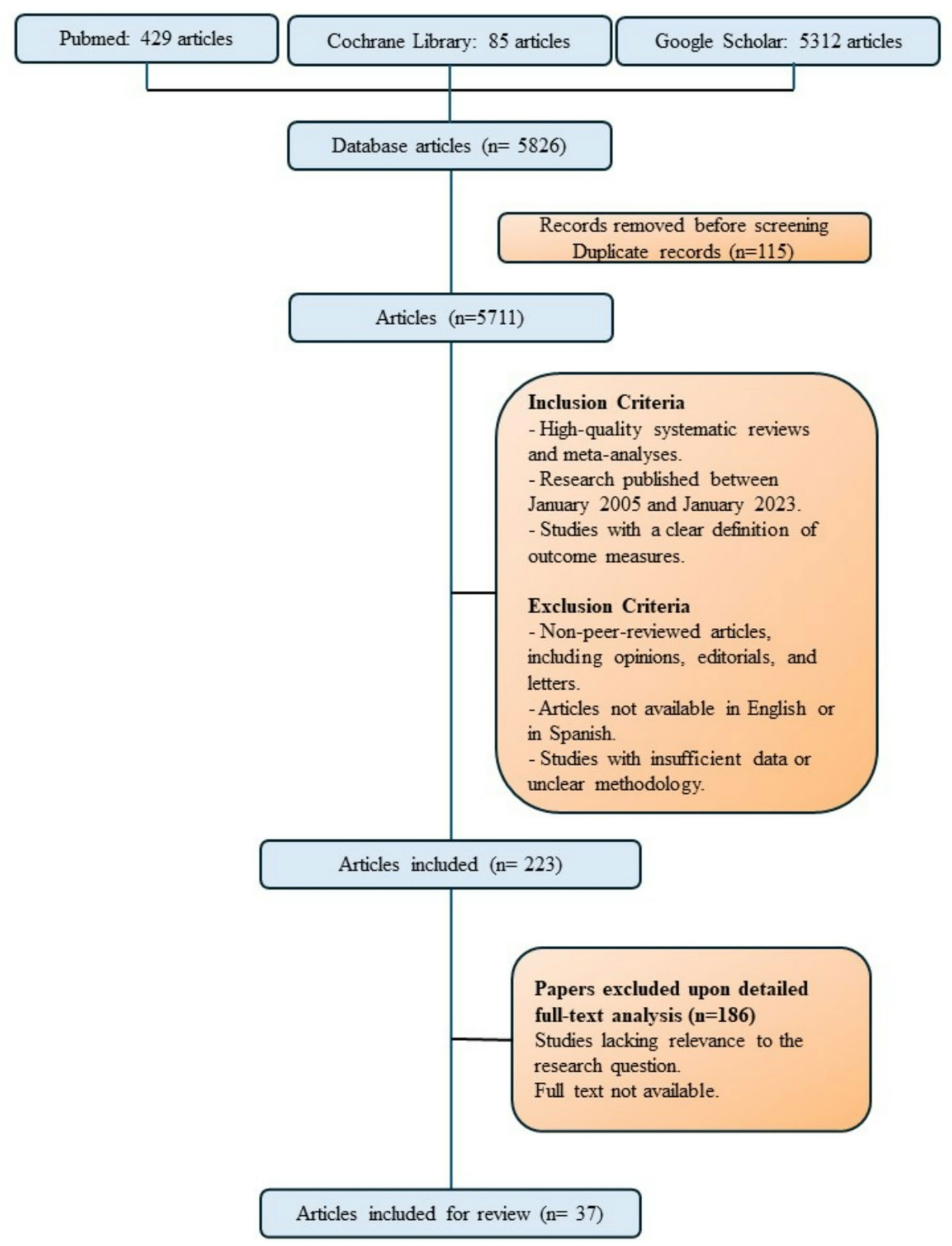
Search flow chart.

A comprehensive bibliographic search was conducted in PubMed, Google Scholar, and the Cochrane Library for articles published between January 2005 and January 2023. The search strategy employed MeSH keywords such as ‘Oropharynx,’ ‘Carcinoma,’ ‘Computerized Tomography,’ and ‘Magnetic Resonance Imaging’, as detailed in Fig. 1. The initial selection process, which was based on predefined scientific quality and inclusion criteria, yielded potentially relevant articles that provided data on key aspects of image interpretation. These articles were subsequently reviewed by three of the authors—two otorhinolaryngologists and one radiologist—who selected key items through three rigorous rounds of review. In these rounds, the remaining authors analysed the revisions that met the stringent inclusion and exclusion criteria. Each record was independently reviewed to identify the most recurrent and significant concepts, specifically those mentioned in international guidelines for the staging and treatment of oropharyngeal tumors and those aligned with the international TNM classification^15^ These criteria formed the basis for a second, more detailed review phase, which focused on including high-quality meta-analyses and systematic reviews for their robust scientific evidence. Additionally, observational studies, selected reference books, and clinical practice guidelines were also considered. Ultimately, these articles were included in the final review, offering a comprehensive overview of the primary concepts to be considered. These sources were meticulously chosen through manual selection and mutual agreement among the authors to ensure comprehensive evidence for the proposed concepts.

### Development of the interface within a mobile application

A web application was developed via the GoodBarber platform, which incorporates the protocol and provides open and free access for users to explore and complete a guided checklist. The application generates detailed summaries while ensuring that no identifiable patient information is collected.

### Study design

This project is integrated into a concordance study designed to evaluate the accuracy of a tool used for radiological image interpretation among radiologists and ENTs. To achieve this goal, a cohort of ENTs was exposed to a randomized series of OPSCC tumor images in two phases: initially without the checklist and subsequently with the checklist.

To undertake this process, radiological images from patients diagnosed with OPSCC were meticulously identified, encompassing those treated in hospitals within the health jurisdictions of the Spanish National Health System in the Valencian Community (specifically, Elda, Alicante, Valencia, and San Juan de Alicante). All patients included in the study were managed and treated in accordance with established clinical practice guidelines. The selected images adhered to specific inclusion criteria: CT and MRI scans of patients diagnosed with OPSCC between 2010 and 2023 systematically managed via a picture archiving and communication system (PACS). CT scans were acquired via multidetector systems with a slice thickness of 2 mm and an overlap of 1 mm to optimize the spatial resolution. Intravenous contrast agent was administered at a dose of 0.5 mg of iodine per kilogram of patient body weight. The MRI protocols included T1-weighted sequences before and after contrast agent administration, T2-weighted sequences for detailed soft tissue assessment, and diffusion-weighted imaging for identifying subtle pathological changes. Imaging acquisition was carefully standardized to minimize artifacts, including motion and metal-induced distortions, by employing advanced reconstruction algorithms and ensuring patient immobilization. All imaging protocols adhered to the quality standards outlined by the European Society of Radiology (ESR) guidelines. The exclusion criteria included images without reports from certified radiology specialists, incomplete images, and cases of OPSCC with tumors extending across multiple regions. Additionally, a cohort of collaborators, consisting of ENT specialists and medical residents who had completed at least three years of residency and were currently affiliated with hospitals within the national health system, was recruited. ENT specialists who were retired or in the process of retirement were excluded from this cohort.

The selected collaborators individually received the images according to a randomized, blind, and independent sampling scheme. Demographic and clinical information, including patient age, sex, and HPV status, was provided in anonymized format on a paper document linked to a unique code assigned to each patient, ensuring strict adherence to confidentiality protocols.

During the first phase, the collaborators reviewed three image series from three oncology patients diagnosed with OPSCC, generating a free-text report and completing the TNM classification. The second phase was conducted exactly seven days after the initial evaluation to minimize recall bias. In this phase, the collaborators evaluated three additional and distinct image series, following the same assessment procedure as in the first phase, with the sole difference being the utilization of the developed checklist during the analysis. Access to the application holding the checklist was granted via a QR code provided to collaborators for use on their own smartphones. Smartphones were required to have the latest version of the software; if this was not feasible, a device was provided to ensure proper access.

In total, each examiner reviewed six distinct cases across the two phases. No feedback or corrective input was provided to the collaborators after the initial evaluations. All the images were reviewed on calibrated 27-inch diagnostic monitors with a resolution of 2560 × 1440 pixels under standardized lighting conditions, ensuring consistency and optimal visualization. Descriptive data were meticulously collected for both the collaborators and the patients.

### Statistical analysis

The analyses were conducted via SPSS version 28 and R version 4.3.1. Initially, a descriptive analysis of all the variables was conducted. Frequencies were calculated for qualitative variables, whereas minimum, maximum, and mean values and standard deviations were determined for quantitative variables. The normality of the data was assessed via the Kolmogorov–Smirnov test. To evaluate the homogeneity of the selected group of collaborators, contingency tables were constructed, and the chi-square test was employed for qualitative variables. For quantitative variables, the mean values were compared via Student’s t test.

To assess whether the tool improved image interpretation, a concordance analysis was conducted. Each item of the tool was treated as a dichotomous variable, with accuracy (yes/no) calculated for each response variable. A “yes” response was assigned when there was concordance between the radiologist and the ENT specialist, and a “no” response was given otherwise. Additionally, precision in the “T” and “N” categories of the international TNM system was evaluated. Finally, an analysis was performed to assess the profile of nonconcordance via a multivariate logistic regression model.

For the concordance analysis between ENT specialists and radiologists, Cohen’s kappa coefficient was estimated for each item, along with its 95% confidence interval. To compare the average concordance effect between the two groups, a meta-analysis approach was employed, including the calculation of a forest plot. This involved determining the average effect for each group, using each item as the unit of analysis and the kappa coefficient for each item as the effect size. The effect between the two groups was analysed by fitting a mixed-effects model, with the group as a moderating variable. Additionally, the number of correct responses for each item in each group was analysed by constructing contingency tables to compare the proportions of correct responses across groups, and the chi-square test was applied. Furthermore, the total number of correct responses across all the items was calculated, and the average number of correct responses in each group was compared via Student’s t test.

## Results

### Review results

Within the context of our study, Fig. 1 serves as a visual representation of the systematic search process and its successive stages. Initially, duplicate articles were removed, yielding a total of 5,711 unique articles. The selected articles were then rigorously evaluated against predefined inclusion criteria, reducing the pool to 223 studies. Following this, several rounds of detailed assessment were conducted to further refine the selection, ultimately identifying 36 articles of the highest relevance and scientific rigor. On the basis of the synthesized data and critical references, a streamlined tool for image analysis was subsequently developed. To facilitate a comprehensive understanding of each concept reviewed, explanatory images were generated, offering visual reinforcement and detailed annotations for each analysed item. (Supplementary material 1.1)

### Key elements for assessing head and neck cancer radiographs: Building our tool

After the evaluation and assessment of key points, a system of pivotal points that may significantly influence the diagnosis, staging, and prognosis of our patients was devised. Additionally, the checklist must be answered with dichotomous or multiple-choice responses to ensure that the user analyses each item for potential involvement. Initially, the checklist user will be prompted to sublocalize the tumor within the area, followed by the initiation of the study of each point^10–12^ These items and the protocol are meticulously distributed and substantiated in the following sections and in Table 1:

- Size: The assessment of a lesion’s size enables its classification according to the current TNM, which is essential for the staging and prognosis of the patient. The user of the checklist must choose among these three options according to the AJCC/UICC classification: size of 2 cm or less, tumors of up to 4 cm, and more than 4 cm^15,16^.
- Extrinsic muscles of the tongue: Extrinsic muscle involvement in tongue tumors informs treatment decisions, classifying them as T4 lesions (T4a in HPV-negative or indeterminate cases)^15,17–19^. After receiving an explanation regarding its identification and exposition of associated implications, the user will proceed to select from a closed-ended dichotomous response (yes/no).
- Crossing of the lingual septum: Drawing an imaginary line through the lingual septum aids in identifying features. Tumors extending across the lingual septum may involve the lingual pedicle, genioglossus muscle, or contralateral half of the tongue^16,18^ Given its implications for prognosis and treatment, the user will analyse this item and respond with yes/no.
- Base of the tongue midline crossing: Identifying this feature is crucial for potential complete excision via transoral robotic surgery (TORS), despite relative contraindications due to potential complications, including swallowing, respiratory, and voice issues, as well as dry mouth^20,21^ It is recommended to mark the midline, provide an example image, and respond yes/no to indicate involvement.
- Involvement of the internal carotid artery: The checklist user will be required to identify the discontinuity of vessel walls or encasement of the internal carotid artery by the tumor under analysis and respond with a dichotomous yes/no answer^22,23^.
- Bone erosion: Cortical bone invasion is characterized by interruption or erosion of the peripheral border on CT or the hypointense border on MRI across all sequences. Attention should be given to the involvement of the mandible, skull base, or hard palate^15,24,25^.
- Perineural dissemination (PD). Imaging signs of PD are highly specific, and some are identified through indirect evidence. Patients with perineural invasion have an increased risk of nodal metastases^26,27^ The nerves involved in this area are the maxillary nerve (V2) and the mandibular nerve (V3), which must be analysed and assessed via dichotomous yes/no responses according to their implications.
- Involvement of the lingual surface of the epiglottis: The spread of primary tumors from the base of the tongue and vallecula to this area does not indicate laryngeal invasion, but identifying it is essential for accurate TNM classification^15,28^ As with the previous items, after the explanation, the user will be asked to respond with a yes/no answer.
- Parapharyngeal and retropharyngeal space. The parapharyngeal space is described as an inverted pyramid extending from the skull base to the hyoid bone’s greater horn. Lesions in the posterior retropharyngeal space necessitate treatment with transoral surgery techniques^20,29^ Given its surgical implications, its identification is essential and requires marking its involvement with a yes/no response.
- Dissemination to the larynx. Dissemination to this region entails the involvement of the supraglottis, glottis, or subglottis. TORS and transoral laser microsurgery (TLM) may provide a viable alternative to open surgery in the salvage setting^15,30^ The user will be asked to analyse the larynx for TNM determination and select the yes/no response accordingly.
- Masticator and nasopharyngeal space. The masticator space houses the four muscles of mastication, ramus, mandibular and maxillary vessels, and nerves (V3 and V2), among others. Nasopharyngeal space involvement often precedes extension to the masticator space^31–33^Tumor involvement of the medial pterygoid muscle or pterygoid plates was classified as T4/T4b^15^. Both spaces must be identified and marked on the checklist with a yes/no response.
- Lymph node dissemination. Approximately 65% of OPSCC patients exhibit cervical lymph node involvement, notably from the tongue base^33,34^The user will be requested to provide the numerical classification for the “N” component of the TNM staging system^15^, following the classification of HPV status as positive, negative, or indeterminate.

**Table 1:** Key items for radiological assessment of oropharyngeal cancers.

Item	Radiological test^6–9^	Key information	Implications for surgical treatment	TNM classification^15^
Size^15,16^	MRI Multidetector CT	Essential for prognosis. Worse prognosis in HPV − tumors	The larger the size, the greater the probability of having to do reconstruction	≤ 2 cm: classified as T1. 2.1–4 cm: classified as T2 > 4 cm: classified as T3
Extrinsic muscles of the tongue^17–19^	Preferentially MRI	Involvement of genioglossus entails a high probability of crossing the lingual septum due to its proximity. Evaluation of floor of mouth involvement, mylohyoid	Predicts the probability of needing neck dissection	T4 HPV+ T4_a_ HPV − or indeterminate
Crossing of the lingual septum^16,18^	Preferentially MRI, contrast-enhanced T1 sequences and T2 sequences Better in coronal slices	Marking of midline. Prognostic indicator for nodal dissemination patterns and impacts survival rates.	Its involvement precludes partial glossectomy and hemiglossectomy. Reconstruction should be considered with flaps.	(Involvement of extrinsic musculature) T4 HPV+ T4_a_ HPV − or indeterminate
Midline crossing (lingual tonsil) ^20,21^	Preferentially MRI, contrast-enhanced T1 sequences and T2 sequences (coronal slice)	Marking of midline. The risk of bilateral metastasis approaches 30%.	Usually, a relative contraindication to surgery. Indication for transoral surgery	
Involvement of the internal carotid^22,23^	Preferentially MRI, contrast-enhanced T1 sequences and T2 sequences Axial slice. Intraluminal tumor is highly specific	Involvement > 270° is associated with embedding and has a mortality rate nearing 100%. If contact affects < 180°, the likelihood of vascular embedding decreases	In case of surgery, ligation is recommended, although due to the prognosis it is not usually done	T4_b_ in HPV − or indeterminate
Bone erosion^24,25^	CT better for cortical bone MRI better for medullar bone	Evaluate mandible, hard palate, and skull base	Early identification will allow marginal or partial mandibulectomy	T4 HPV+ T4_a_ HPV − or indeterminate. In case of involvement of skull base: T4_b_ in HPV– or indeterminate
Perineural dissemination^26,27^	Preferentially MRI	The obliteration of the fatty plane surrounding the cranial nerve, enhancement with or without thickening of the nerve, and the denervation of a group of muscles innervated by the cranial nerve^26^	15–20% lymph node involvement. Propose cervical dissection according to guidelines	
Involvement of the lingual surface of the epiglottis^28^	Preferentially MRI, contrast-enhanced T1 sequences and T2 sequences Multidetector CT. Sagittal section	Not to be confused with laryngeal involvement	Requires excision of epiglottis; risk of aspirations	T3
Parapharyngeal and retropharyngeal space^20,29^	Preferentially MRI, contrast-enhanced T1 sequences and T2 sequences	Virtual space, usually made up of fat. Implies the involvement of the constrictor muscles	Accessible through transoral surgery like TORS	
Dissemination to the larynx^30^	Preferentially multidetector CT	Involvement of the epiglottis in tumors at the base of the tongue increases the risk o	The resection could be extended without modifying the field in TORS + TLM	T4 HPV+ T4_a_ HPV − or indeterminate
Masticator space^31,32^	Sequences in T1 with contrast and T2	Identify lateral pterygoid, medial pterygoid, and pterygoid plates^31^	Very complex, complete resection by transoral techniques	In case of involvement of lateral pterygoid and pterygoid plates T4_b_ in HPV– or indeterminate o T4 in HPV+.
Nasopharyngeal space^33^	Contrast-enhanced T1 sequences and T2 sequences	Special attention to the lateral wall	More complex excision, requires multiple techniques	T4_b_ in HPV– or indeterminate
Lymph node dissemination^33,34^	MRI, contrast-enhanced T1 sequences and T2 sequences PET-CT Multidetector CT Ultrasound (experienced user)	High incidence > 15 mm in jugulodigastric lymph nodes and > 10 mm in other lymph nodes. Cystic adenopathy with a hypodense center indicates HPV-positive SCC	Investigate areas I (base of the tongue), II, III and IV (tonsils)	Classification N

Upon analysis of all the items, the user will be requested to provide a numerical classification for the “T” component of the TNM system^15^, utilizing a table that outlines each area of involvement corresponding to each number. Ultimately, the user will receive a summary of their responses, identified by a code they specified at the beginning of the checklist.

### Study concordance results

The results of this study were initiated by the creation of an image repository comprising 45 groups of patients diagnosed with OPSCC, carefully selected on the basis of strict inclusion and exclusion criteria. Following a thorough randomization process, a total of 90 image groups were analysed across two distinct phases (see Table 2). The statistical analysis of the variables yielded p values > 0.001 in all instances, suggesting homogeneity among the groups and indicating that the observed variations are due to random chance rather than inherent differences between the groups. This finding supports the comparability and consistency of the data, thereby reinforcing the validity of the study’s results.

**Table 2:** Contingency table for the descriptive analysis of the two study groups.

		Without checklist		With checklist		*p*-valor
		*n*	%	*n*	%	
Origin	Alicante	30	66,7%	31	68,9%	0,822
	Valencia	15	33,3%	14	31,1%	
Sex	Female	16	35,6%	10	22,2%	0,163
	Male	29	64,4%	35	77,8%	
Stage at Diagnosis	1	7	15,6%	4	8,9%	0,563
	2	3	6,7%	2	4,4%	
	3	11	24,4%	8	17,8%	
	4	0	0,0%	3	6,7%	
	4a	14	31,1%	17	37,8%	
	4b	7	15,6%	8	17,8%	
	4c	3	6,7%	3	6,7%	
Metastasis	0 (No metastasis)	42	93,3%	42	93,3%	1,000
	1(Metastasis)	3	6,7%	3	6,7%	
HPV	Negative/Undetermined	32	71,1%	35	77,8%	0,468
	Positive	13	28,9%	10	22,2%	
Age	Media (SD)	59,4	(10,3)	62,3	(9,5)	0,179

**Fig. 2:**
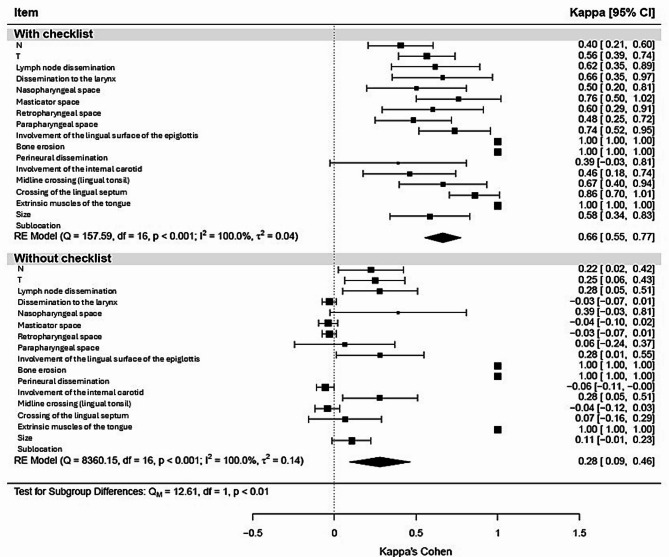
Concordance analysis for each item within each group.

The images were evaluated by a group of 15 collaborators selected according to predefined inclusion criteria. The concordance between ENT specialists and radiologists was analysed via a meta-analysis approach (Fig. 2). Since all the items involved the same subjects, the overall effect was not weighted. For items demonstrating total concordance, the kappa coefficient was set to 1 to be included in the calculation of the overall effect. The forest plot, which includes subgroup analyses, was generated by fitting a mixed-effects model and conducting a heterogeneity analysis. The Cohen’s kappa coefficient for the checklist group (0.66, 95% CI: 0.55–0.77) was significantly greater than that for the nonchecklist group (0.28, 95% CI: 0.09–0.46), with a p value for the group difference of < 0.01.

The analysis of the data in Table 3 revealed a greater proportion of correct responses in the checklist group for the items “sublocalization,” “size,” “musculature,” and “adenopathy.” The mean total number of correct responses across all the items was significantly greater in the checklist group (14.6) than in the nonchecklist group (12.0), within a possible range of 0–17 correct responses.

**Table 3:** Analysis of correct answers for each item by group.

		Without checklist		With checklist		
		*n*	%	*n*	%	*p*-valor
Sublocation	Incorrect	27	60,0%	8	17,8%	< 0,001
	Correct	18	40,0%	37	82,2%	
Size	Incorrect	39	86,7%	0	0,0%	< 0,001
	Correct	6	13,3%	45	100,0%	
Extrinsic muscles of the tongue	Incorrect	17	37,8%	3	6,7%	< 0,001
	Correct	28	62,2%	42	93,3%	
Crossing of the lingual septum	Incorrect	8	17,8%	5	11,1%	0,368
	Correct	37	82,2%	40	88,9%	
Midline crossing (lingual tonsil)	Incorrect	15	33,3%	10	22,2%	0,239
	Correct	30	66,7%	35	77,8%	
Involvement of the internal carotid	Incorrect	4	8,9%	4	8,9%	1,000
	Correct	41	91,1%	41	91,1%	
Perineural dissemination	Incorrect	45	100,0%	45	100,0%	-
Bone erosion	Incorrect	0	0,0%	1	2,2%	0,315
	Correct	45	100,0%	44	97,8%	
Involvement of the lingual surface of the epiglottis	Incorrect	12	26,7%	5	11,1%	0,059
	Correct	33	73,3%	40	88,9%	
Parapharyngeal space	Incorrect	13	28,9%	12	26,7%	0,814
	Correct	32	71,1%	33	73,3%	
Retropharyngeal space	Incorrect	3	6,7%	5	11,1%	0,459
	Correct	42	93,3%	40	88,9%	
Masticator space	Incorrect	5	11,1%	3	6,7%	0,459
	Correct	40	88,9%	42	93,3%	
Nasopharyngeal space	Incorrect	5	11,1%	7	15,6%	0,535
	Correct	40	88,9%	38	84,4%	
Dissemination to the larynx	Incorrect	3	6,7%	4	8,9%	0,694
	Correct	42	93,3%	41	91,1%	
Lymph node dissemination	Incorrect	15	33,3%	6	13,3%	0,025
	Correct	30	66,7%	39	86,7%	
T	Incorrect	28	62,2%	15	33,3%	0,006
	Correct	17	37,8%	30	66,7%	
N	Incorrect	29	64,4%	22	48,9%	0,136
	Correct	16	35,6%	23	51,1%	
Total number of correct answers (0–17) *	Media (SD)	12,0	(1,7)	14,6	(1,5)	< 0,001

*Normality test for the total number of correct answers: *p* = 0,097.

Finally, a mixed-effects linear model with a random effect for patients revealed that the checklist group had a mean of 2.66 more correct responses than the nonchecklist group did, with a standard error of 0.31 and a p value < 0.001, adjusted for sex, age, stage, and HPV status.

## Discussion and conclusions

The results of the study of the developed tool revealed a significant increase in the kappa index when the two groups were compared, highlighting a clinically and statistically meaningful improvement in accuracy and reliability when the tool was utilized by ENT specialists in clinical practice. With the checklist, the kappa value reached 0.66, indicating a proficient level of agreement, compared with 0.28 without the checklist, which reflects only slight or minimal agreement. This increase in kappa indices suggests greater consistency and concordance between the assessments made by ENT specialists and radiologists, thereby underscoring the efficacy and utility of the tool as a complementary resource. Furthermore, the analysis of the mean total number of correct responses for all the items revealed a significantly higher score in the group that used the checklist. This finding underscores the tool’s overall effectiveness in enhancing diagnostic accuracy. A mixed-effects linear model with a random effect for patients, adjusted for variables such as sex, age, stage, and HPV status, provided further evidence of the checklist’s utility, even when additional factors that could influence the results were considered.

To facilitate the implementation of the checklist, a mobile application titled NECKCHECK was developed and is accessible at https://neckcheck.goodbarber.app/ (Supplementary material 1.2). This software provides an interactive platform for the interpretation and assessment of radiological images of OPSCC, thereby enhancing the accessibility and standardization of the diagnostic process. Similar systems are frequently employed in routine clinical practice. A randomized controlled trial investigating an educational intervention in radiology, using a comparable web-based application hosted on Radiopaedia.org, reported an improvement in radiological knowledge test scores in the group that used the system compared with those taught by traditional methods^35^ Similar software with high acceptance among physicians, such as CTisus (https://www.ctisus.com), a specialized radiology platform that provides support for image interpretation and educational materials, has been studied and analysed^36^ Similarly, the Radiology Assistant (https://radiologyassistant.nl), developed by the Dutch Society of Radiology, offers up-to-date and peer-reviewed radiological cases and articles^37^ NECKCHECK offers similar benefits by applying radiological and clinical concepts to the management of SCC.

Scientific evidence indicates that checklists improve the detection of pathologies by providing a structured framework, even when employed by experts in the field^38^ These tools ensure that all relevant aspects of a pathology are systematically evaluated. Checklists standardize the assessment process, facilitate training, enhance multidisciplinary communication, and reduce diagnostic errors. They have been effectively utilized in various medical domains, including research evaluation through initiatives such as STARD^39^ and the WHO Surgical Safety Checklist, which has demonstrated a reduction in perioperative errors by decreasing the incidence of complications and mortality^40^ In the field of otolaryngology, this approach is analogous to the 16-point radiological checklist developed by Mather et al.^41^ for assessing acute mastoiditis and its complications, as well as the checklist created by Maza-Lozano et al. for interpreting radiological images that encompass nasosinus anatomical structures. This latter checklist aids in the identification of critical landmarks, thereby facilitating systematic and preoperative planning for surgical procedures^42^.

Currently, significant emphasis in cancer diagnosis has been placed on the analysis and processing of digital images. This approach involves extracting meaningful information from these images to delineate clinically relevant features or to classify them accordingly. Machine learning techniques based on feature analysis have demonstrated efficacy in various diagnostic applications by explicitly defining a predetermined set of features and processing steps^43^ The development of a system for identifying key points or “red flags,” as exemplified by the tool created in this study, could be integrated into artificial intelligence systems. Despite the rapid and ongoing development of such systems, they have yet to reach a level of clinical usability in most predictive models^44^.

This application, although limited to an algorithmic response based on a checklist, represents a significant contribution to the project. By consolidating this functionality into a single web application, it simplifies the workflow and enhances the user experience, thereby increasing its utility and relevance in the clinical setting. The strength of this system lies in its ability to provide a systematic framework for image evaluation, helping with clinical data collection and standardizing the analysis process. This reduces interobserver variability and ensures that all specialists adhere to the same evaluation criteria. This feature offers an effective solution to address the complexity of evaluating these tumors, supporting ongoing medical education for specialists and promoting evidence-based practice. Overall, the checklist has shown favourable results, with high-performing items identified, such as “sublocalization”, “size”, “extrinsic musculature” and “adenopathy.” However, there is room for improvement in other items, which carry minimal weight within the tool, as indicated by independent area analysis. To further enhance the kappa index and achieve higher levels of reliability, more detailed explanations or additional images that clarify concepts more precisely could be incorporated. Additionally, removing items with minimal impact on the tool’s overall effectiveness may also contribute to improved outcomes.

Although the study results demonstrated a high kappa index, it is essential to assess whether this level of performance is sufficient for the checklist to replace the radiologist at its current stage of development. While the checklist effectively enhances the identification of diagnostic elements, it should initially be regarded as a complementary and educational tool. Its potential influence on clinical and therapeutic decisions should be approached cautiously and used in conjunction with expert radiological interpretation, underscoring the indispensable role of radiologists in comprehensive diagnosis and treatment planning.

In summary, our findings support the effectiveness of the digital checklist as an educational and useful tool that enhances consistency in the evaluation of OPSCC images by ENT specialists. The developed system provides a notable benefit by improving accuracy in the interpretation of OPSCC images, even when accounting for potential confounding variables. Nevertheless, these results should be interpreted with caution and further corroborated by additional research conducted in diverse clinical environments.

## Electronic supplementary material

Below is the link to the electronic supplementary material.

Supplementary Material 1

## Data Availability

The data supporting the findings of this study are available through the NECKCHECK PROJECT: Enhancing diagnostic accuracy in oropharyngeal squamous cell carcinoma through computer-based radiological tools, which can be accessed via Figshare at https://doi.org/10.6084/m9.figshare.26928577.v1.

## Abbreviations

CT: Computed tomography
HPV: Human papillomavirus
MRI: Magnetic resonance imaging
PET: Positron emission tomography
TORS: Transoral robotic surgery
TLM: Transoral laser microsurgery
SCC: Squamous cell carcinoma
ESR: European Society of Radiology
