# Supplementary material for: NECKCHECK PROJECT: enhancing diagnostic accuracy in oropharyngeal squamous cell carcinoma through computer-based radiological tools
Source: Sci Rep. 2025 Jun 4;15:19645. doi: 10.1038/s41598-025-03895-8 (PMC12137671; doi:10.1038/s41598-025-03895-8)

**Supplementary material 1.1**

Model images


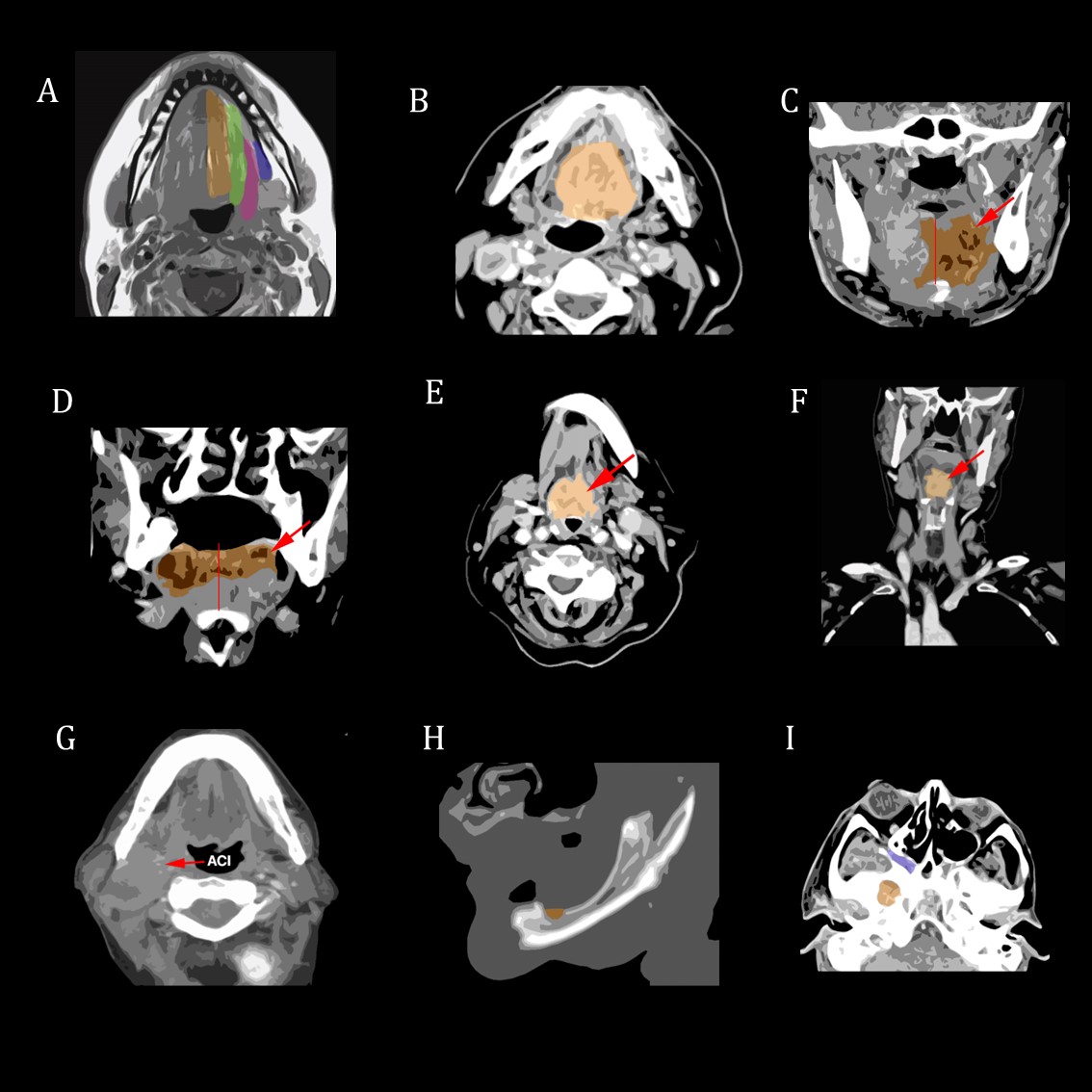


A. Axial section. In orange, Genioglossus muscle; in green, Hyoglossus muscle; in pink, Styloglossus muscle; and in purple, Mylohyoid muscle.

B. Axial section. Tumor in orange infiltrating the lingual musculature.

C and D: Coronal section. The red arrow indicates the tumor surpassing the lingual septum. The red line is drawn as an example for identifying the midline

E. Coronal Section: Illustration showing the tumor affecting both sides of the midline.

F. Axial Section: Illustration depicting the tumor crossing the midline and infiltrating surrounding structures.

G. Axial section showing a lymph node conglomerate infiltrating or encasing the internal carotid artery by more than 270 degrees.

H. Sagittal section. Bone erosion in the mandible highlighted in orange

I. Axial section. Identification of the Maxillary nerve (V2) in green as it exits the foramen rotundum and Mandibular nerve (V3) in brown. The pterygopalatine fossa is depicted in purple.


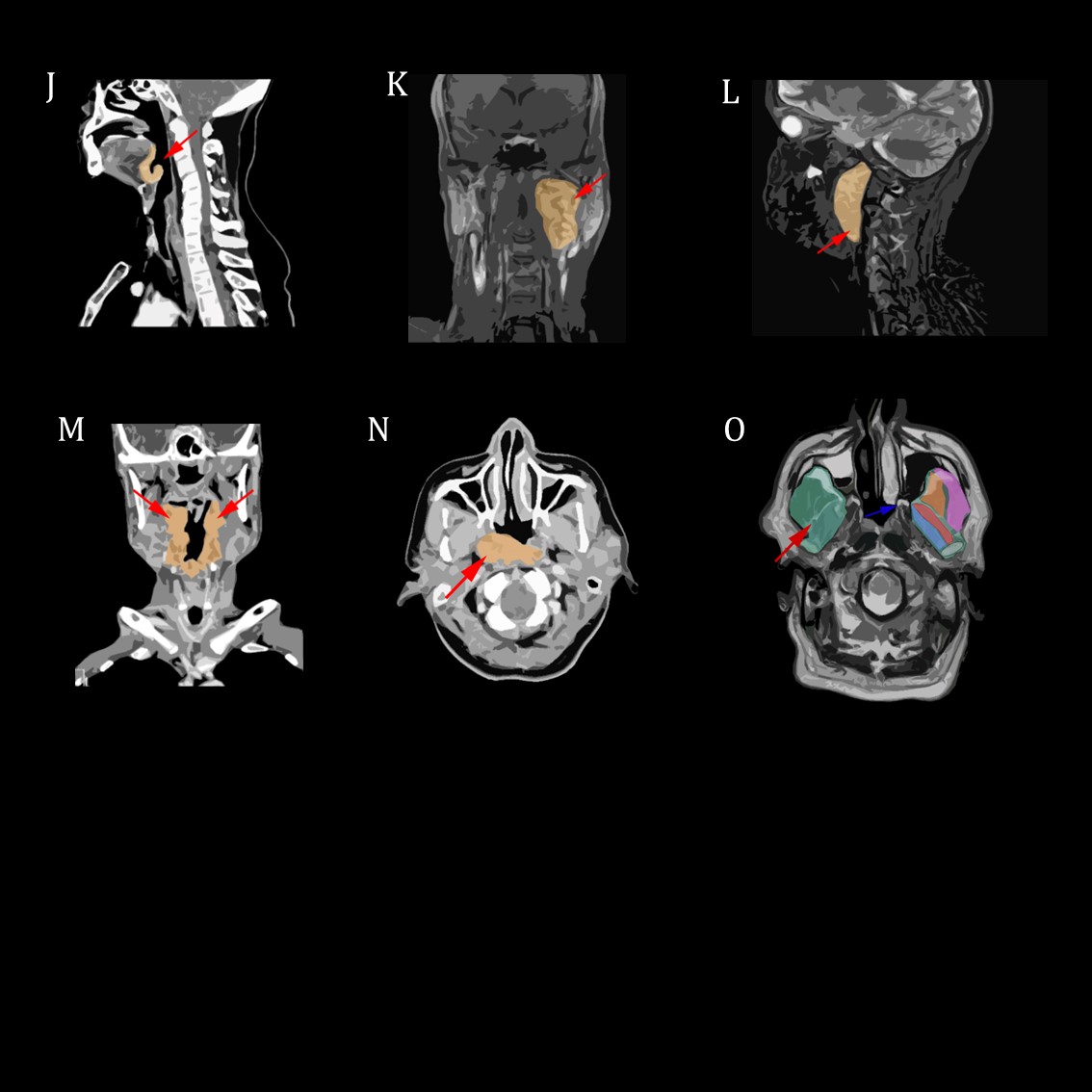


J. Corte sagital. Erosión ósea en naranja de la mandíbula

K. Sagittal section. Oropharyngeal tumor affecting the lingual surface of the epiglottis in Orange

L (Sagittal Section) and M (Coronal Section). The red arrow indicates a tumor affecting the left Parapharyngeal space.

N. (Axial section) The red arrow indicates a tumor with involvement of the nasopharynx (orange).

O. (Axial section) In green: Masticator space; In blue: Medial Pterygoid Muscle; In red: Lateral Pterygoid Muscle; In pink: Masseter Muscle; In orange: Temporal Muscle; Blue arrow: Pterygoid Process; Red arrow: Inferior Alveolar Vessels.

**Supplementary material 1.2**

Web application


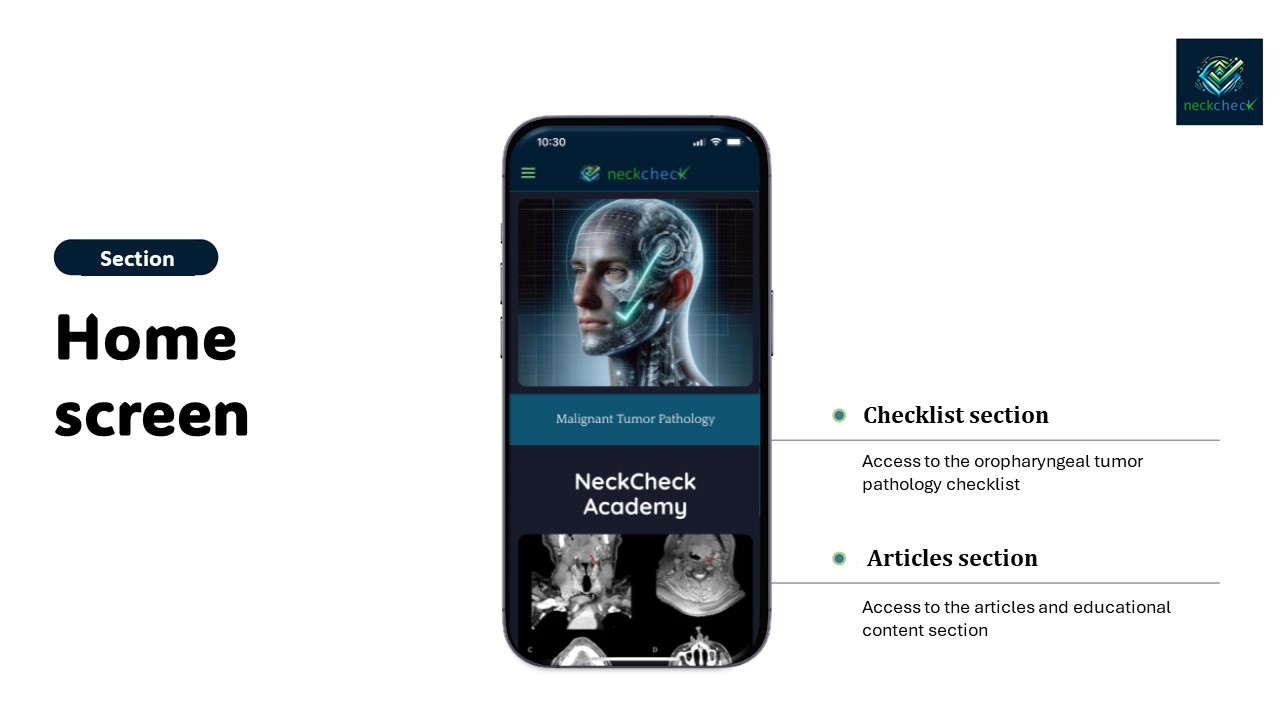


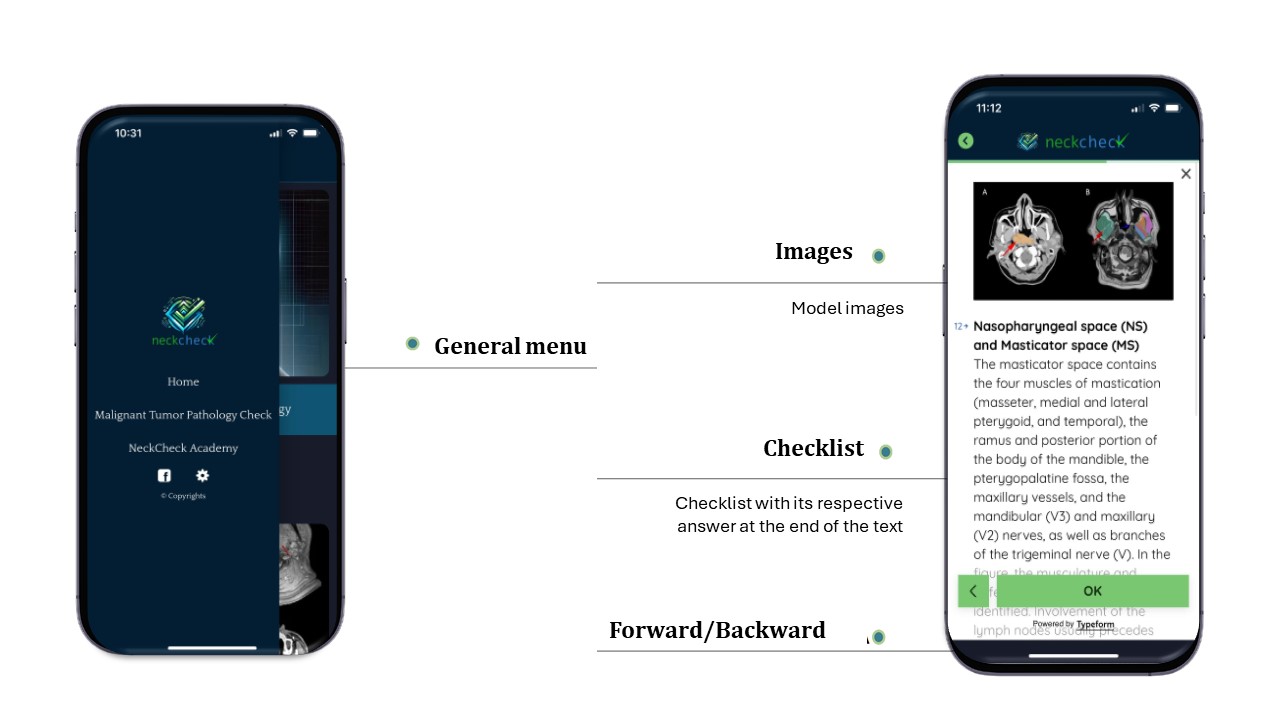


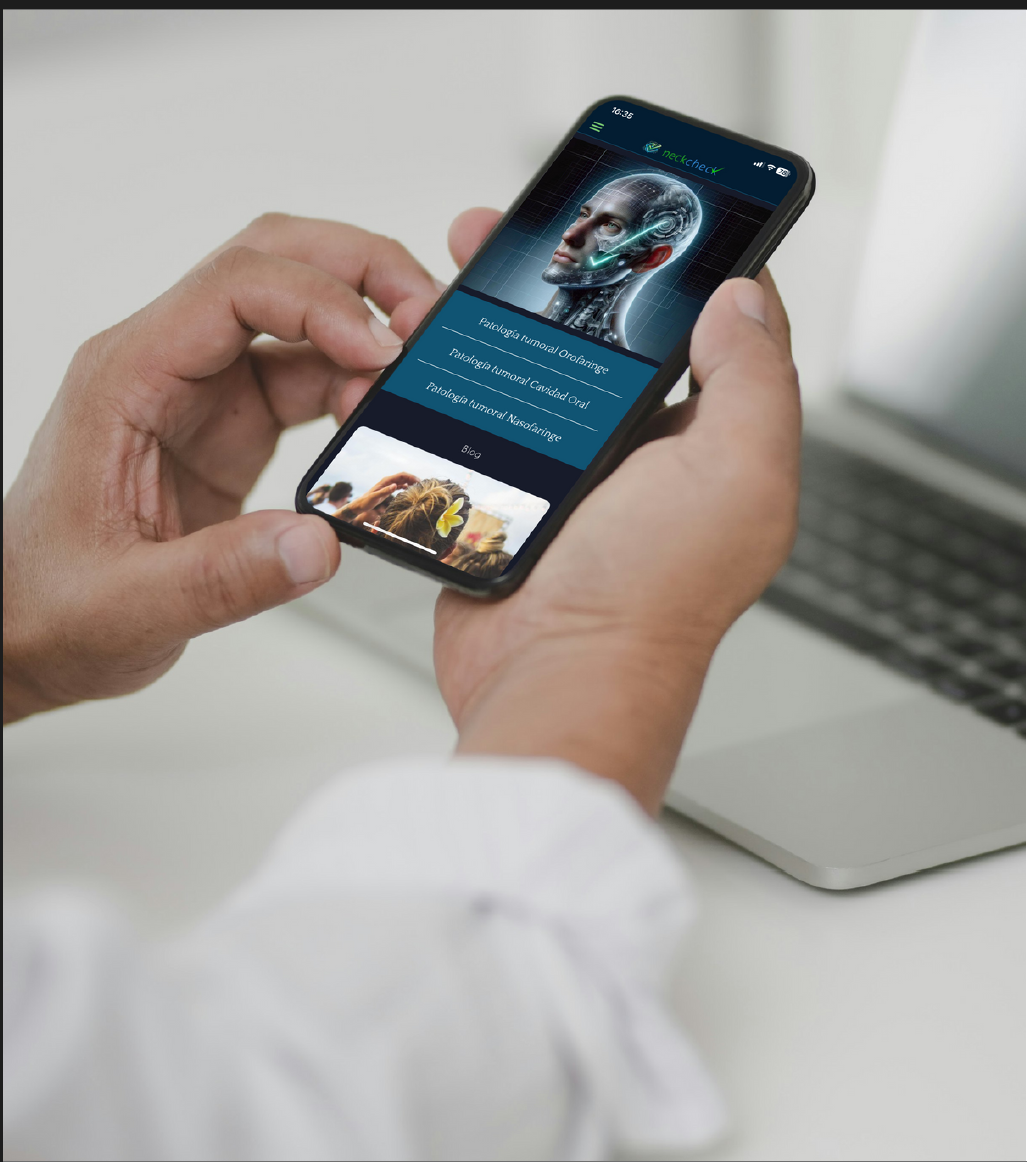

Supplement: Supplementary file 1 — Supplementary Material 1 [file 41598_2025_3895_MOESM1_ESM.docx]
